# Preferences Regarding Information Strategies for Digital Mental Health Interventions Among Medical Students: Discrete Choice Experiment

**DOI:** 10.2196/55921

**Published:** 2024-10-04

**Authors:** Markus Vomhof, Jessica Tabea Bau, Pia Hüter, Stefan Stehl, Burkhard Haastert, Adrian Loerbroks, Andrea Icks, Stella Teresa Calo, Luca Schuster, Claudia R Pischke, Nadja Kairies-Schwarz, Peter Angerer, Jennifer Apolinário-Hagen

**Affiliations:** 1 Institute for Health Services Research and Health Economics Centre for Health and Society, Medical Faculty and University Hospital Düsseldorf Heinrich Heine University Düsseldorf Düsseldorf Germany; 2 Institute for Health Services Research and Health Economics German Diabetes Center, Leibniz Center for Diabetes Research Heinrich Heine University Düsseldorf Düsseldorf Germany; 3 Institute of Medical Sociology Centre for Health and Society, Medical Faculty and University Hospital Düsseldorf Heinrich Heine University Düsseldorf Düsseldorf Germany; 4 Institute of Occupational, Social and Environmental Medicine Centre for Health and Society, Medical Faculty and University Hospital Düsseldorf Heinrich Heine University Düsseldorf Düsseldorf Germany; 5 mediStatistica Wuppertal Germany

**Keywords:** preferences, digital mental health, medical students, innovation diffusion, technology acceptance, health information

## Abstract

**Background:**

Digital mental health interventions (DMHIs) are capable of closing gaps in the prevention and therapy of common mental disorders. Despite their proven effectiveness and approval for prescription, use rates remain low. The reasons include a lack of familiarity and knowledge as well as lasting concerns. Medical students were shown to have a comparatively higher risk for common mental disorders and are thus an important target group for raising awareness about DMHIs. At best, knowledge is already imparted during medical school using context-sensitive information strategies. Yet, little is known about medical students’ information preferences regarding DMHIs.

**Objective:**

This study aims to explore information preferences for DMHIs for personal use among medical students in Germany.

**Methods:**

A discrete choice experiment was conducted, which was developed using an exploratory sequential mixed methods research approach. In total, 5 attributes (ie, source, delivery mode, timing, recommendation, and quality criteria), each with 3 to 4 levels, were identified using formative research. Data were analyzed using logistic regression models to estimate preference weights and the relative importance of attributes. To identify subgroups of students varying in information preferences, we additionally performed a latent class analysis.

**Results:**

Of 309 participants, 231 (74.8%) with reliable data were included in the main analysis (women: 217/309, 70.2%; age: mean 24.1, SD 4.0 y). Overall, the conditional logit model revealed that medical students preferred to receive information about DMHIs from the student council and favored being informed via social media early (ie, during their preclinic phase or their freshman week). Recommendations from other students or health professionals were preferred over recommendations from other users or no recommendations at all. Information about the scientific evidence base was the preferred quality criterion. Overall, the timing of information was the most relevant attribute (32.6%). Latent class analysis revealed 2 distinct subgroups. Class 1 preferred to receive extensive information about DMHIs in a seminar, while class 2 wanted to be informed digitally (via email or social media) and as early as possible in their studies.

**Conclusions:**

Medical students reported specific needs and preferences regarding DMHI information provided in medical school. Overall, the timing of information (early in medical education) was considered more important than the information source or delivery mode, which should be prioritized by decision makers (eg, members of faculties of medicine, universities, and ministries of education). Study findings suggest general and subgroup-specific information strategies, which could be implemented in a stepped approach. Easily accessible digital information may promote students’ interest in DMHIs in the first step that might lead to further information-seeking behavior and the attendance of seminars about DMHIs in the second step.

## Introduction

### Background

Despite the considerable burden attributable to common mental disorders (CMDs), numerous structural obstacles to the use of (face-to-face) psychological interventions, such as waiting times, remain [1]. In addition, individual-level barriers, such as skepticism about helpfulness and safety, self-stigma, or simply a lack of awareness of appropriate services, are well documented [2,3]**.** Given the need for lowering the threshold to access psychological interventions, digital mental health interventions (DMHIs) can play a key role [4]. They can be differentiated by their application field (eg, prevention vs therapy), technical modality (eg, app and virtual reality), guidance (varying in source, timing, and intensity; eg, via videoconference) or application area (eg, stand-alone vs blended care), and theory base (eg, cognitive behavioral therapy) [5,6]. With the Digital Healthcare Act, German health policy created new regulations for the certification and reimbursement of costs for prevention and health care contexts. Since October 2020, physicians and psychotherapists are allowed to prescribe specific certified medical smartphone apps and browser-based applications (German: *Digitale Gesundheitsanwendungen* [DiGA]) listed in a repository by the Federal Institute for Drugs and Medical Devices (German: Bundesinstitut für Arzneimittel und Medizinprodukte [BfARM]), including digital therapeutics for different CMDs, on the expense of statutory health insurance companies [7,8]**.** Nevertheless, the general use of DMHIs across European countries in the past years [4] as well as the uptake of DMHIs in terms of DiGA in Germany in recent times is marginal and appears to be hampered by individual-level barriers, such as doubts and lack of knowledge, among health care professionals (HCPs) and patients [9,10]. In addition, the diffusion process is also affected by health policy barriers, such as adverse regulations and increasing barriers to market entry for potential DiGA providers [11]. The distinction between permanent and temporary listing of DiGA has also been criticized, especially by statutory health insurance companies. Although most DiGA in the field of DMHIs are permanently listed based on their scientific evidence base, the so-called fast-track certification of DiGA has raised concerns about the scientific foundation of temporarily listed DiGA. This means that manufacturers that meet the criteria by the BfArM, except for a high-quality randomized controlled trial during the proposal, can get a listing for their DiGA if the concept is likely to succeed. However, it should be considered that manufacturers have an immense financial risk if they fail to prove the positive care effects with rigorously conducted randomized controlled trials within 1 year or if their DiGA price will be upgraded after the test phase. In addition, no DiGA gets a listing by the BfARM, even not provisionally, if no sufficient data for its efficacy from pilot studies was available or data security standards were not met [8]. However, long-term effects and adherence among various patient groups should be monitored, given that the DiGA concept is rather novel. Particularly**,** the outbreak of the COVID-19 pandemic and the accompanying need to quickly switch to contactless health care made it clear that lacking acquaintance with DMHIs is a key barrier to their implementation in health care [12]**.** Therefore, the development of user-centered, context-sensitive information strategies for relevant target groups represents a crucial step during the slow innovation diffusion process [13].

When analyzing preferences regarding information strategies on DMHIs, medical students represent a special population of interest not only because they potentially prescribe DMHIs in their role as future physicians but also belong to a group considered vulnerable in terms of a high prevalence of CMDs and often exhibit poor help-seeking behavior when in need [14-16]. Fear of stigmatization in medical school and expected career disadvantages, if mental health issues are disclosed, appear to be widespread reasons for not seeking support [17].

To inform patients and (future) HCPs about DMHIs, acceptance-facilitating interventions (AFIs) have been suggested as information strategies in earlier stages of innovation diffusion [18]**.** AFIs typically consist of multiple components, such as narrative messages (eg, experts’ recommendations), information on quality criteria, including the scientific evidence base, as well as the use of different media formats ranging from text-based information to video material [19]**.** Besides insignificant results in some studies on the effects of AFIs [20-22], different experiments have shown that AFIs can foster the acceptance of DMHIs among individuals with CMDs [23,24], students with or without mental health issues [25], and psychotherapists [18].

However, the optimal composition of multiattribute AFIs for providing DMHI information is difficult to determine with commonly used survey methods that do not require respondents to make trade-offs between the components they prefer the most. Discrete choice experiments (DCEs) offer the possibility to investigate complex hypothetical choices of information strategies [26] by involving combinations of various information attributes, varying in attribute levels, and controlling for interactions.

To our knowledge, only few DCEs examined information preferences on mental health services (eg, the study by Cunningham et al [27]), but none of them focused on DMHIs. Furthermore, while prior DCEs have focused on preferences regarding the delivery of DMHIs in the general population and among HCPs in Germany (refer to the studies by Phillips et al [28,29]), little is known about the specific information preferences regarding DMHIs in medical students.

### This Study

This DCE aims to examine information preferences on DMHIs for personal use among medical students in Germany. For this purpose, 3 research questions were formulated: (1) What is the preferred information strategy regarding DMHIs (ie, AFIs) in medical students? (2) What are the most important attributes of information strategies? and (3) Does preference heterogeneity exist regarding information strategies?

## Methods

### Ethical Considerations

The ethics committee of the Faculty of Medicine at the Heinrich Heine University Düsseldorf approved the DCE in August 2022 (approval number 2022-2102).

The DCE was conducted web-based and anonymously using Lighthouse Studio (Sawtooth Inc). No identifying information, such as personal data or IP addresses, was recorded to ensure anonymity. To access the web survey, participants had to provide informed consent (click-to-agree). Participants were able to quit the assessment whenever they wanted. As an incentive, participants could take part in a lottery (5 × €100 [US $110.5] and 30 × €50 [US $55.3]) upon study completion by saving a randomly created code consisting of numbers (eg, via screenshot). No contact data were thus requested for participation in the lottery. This ensured the anonymity of participation. The winning codes were published on the website of the institute in May 2023. The payment was handled by university staff not involved in the research.

### Study Design

The presented DCE was the main study of an exploratory mixed methods research approach. Next, we present the development of the DCE. Both study parts have been preregistered [30,31].

Following a sequential methodological approach and recommendations on the construction of DCEs according to the International Society for Pharmacoeconomics and Outcomes Research [32] and Hollin et al [33], the DCE development was grounded in literature research and formative qualitative research.

The development consisted of the following 6 sequential steps: (1) reviewing the research literature (for the research proposal), (2) conducting semistructured qualitative interviews, (3) conducting co-design workshops with medical and psychology students, (4) cognitive interviewing (concurrent think-aloud technique) and selection of attributes, (5) generating the experimental design, and (6) technical pretesting.

### Steps of DCE Development

#### Preliminary Work: Literature Research

Research objectives were defined based on the current literature (using electronic databases and hand searching). In the first step, literature was searched to identify general information needs and preferences regarding DMHIs as well as potential acceptance-facilitating features of information on DMHIs among university students as potential users. This was the basis for the research proposal to be funded by the Research Commission of the Medical Faculty at the Heinrich Heine University Düsseldorf (grant 2020-60). As outlined in the Introduction section, this step led to the decision to focus the study on students of health care–related subjects, and especially medical students. In addition, because of the fragmentary research on the topic, a mixed methods design was chosen. In the second step, findings were used to develop semistructured topic guides for the qualitative interviews.

#### Qualitative Interviews

To explore the specific needs and preferences of students related to health care across Germany, we conducted 21 semistructured individual web-based interviews until consensus and thematic saturation were achieved (16/21, 76% medicine; 5/21, 24% psychology; female: 16/21, 76%; mean age 25.5, SD 3.9 y) in August and September 2021 using videoconference software (Webex; Cisco Systems). The interviews were saved using a digital recorder device and had an average duration of 31.7 (SD 10.3) minutes. The data were analyzed by applying content analysis according to Mayring [34], using MAXQDA, version 2020 (VERBI GmbH). Students indicated little knowledge and experience with DMHIs but positive attitudes toward their potential use. They were asked about their information preferences regarding design (eg, text or video), content (eg, data protection and costs), and source (eg, university or physician). We deductively derived 4 attributes of an information strategy: information source, delivery format, content preferences, and general design preferences, varying in different attribute levels (for a detailed description, refer to the study by Braun et al [35]). The results were used to develop a preliminary set of choice tasks for the DCE to be tested in co-design workshops for comprehensibility, relevance, completeness, and feasibility in the next step [26,32].

The interviews also addressed the setting in which participants would like to receive information about DMHIs. Medical students had preferred the university context in our prior work, which was therefore chosen as a context in the DCE (for detailed information, refer to studies by Braun et al [35] and Dederichs et al [36,37]).

#### Co-Design Workshops

To involve the target group in the DCE design and to validate and refine the selection of attributes and levels, 2 participatory co-design workshops with 8 university students (6/8, 75% medicine; 1/8, 12% psychology; and 1/8, 12% public health) were conducted in May and June 2022. The face-to-face workshops lasted approximately 90 to 120 minutes. Participants were asked to evaluate prepared choice tasks with respect to comprehensibility and cognitive effort. Owing to the workshops, we decided to focus on medical students only in the further preparation and realization of the DCE because the 2 (25%; nonmedical) of the 8 students expressed slightly different preferences for attributes and levels than medical students, which was interpreted in terms of their different study background. Furthermore, we made minor changes to the set of attributes, splitting content preferences into *recommendation* (eg, HCPs and patients) and *quality criterion* (eg, data security and scientific evidence base) and dropping general design preferences because it was rated least important. Furthermore, we added a new attribute called *timing* (eg, freshman week and preclinic), which seemed to be of greater importance to participants.

#### Cognitive Interviews

In July 2022, the preliminary DCE was discussed in cognitive interviews with 5 respondents. Participation required informed consent. Using the concurrent think-aloud method, which is an established approach in usability research [38], respondents were asked to share their thoughts while answering the survey, including DCE tasks and instructions. The piloting lasted approximately 60 minutes per participant and aimed to check again for comprehensibility and to identify difficulties in processing. Gathered information only led to some minor rearrangements.

#### Experimental Design

The experimental design was based on the decision by the research team to choose a set of attributes and levels following the qualitative research steps as well as plausibility considerations on combinations. The choice sets did not include an opt-out option because we wanted to learn which information strategy is preferred by students, given the university would decide to undertake an information campaign for DMHIs. A fractional factorial experimental design [39] was developed based on the study by Kuhlfeld [40] using the statistical software package SAS (SAS Software 9.4 [TS1M2]; SAS Institute Inc). The design allowed for an estimation of the main effects, with all attributes coded categorically. D-efficiency was 16.029. We used effects variable coding according to Bech and Gyrd-Hansen [41]. In the end, the experimental design consisted of 24 choice tasks, which we divided into 8 choice tasks administered in 3 blocks. Each choice set consisted of 5 attributes varying in 3 to 4 attribute levels.

**Figure 1:**
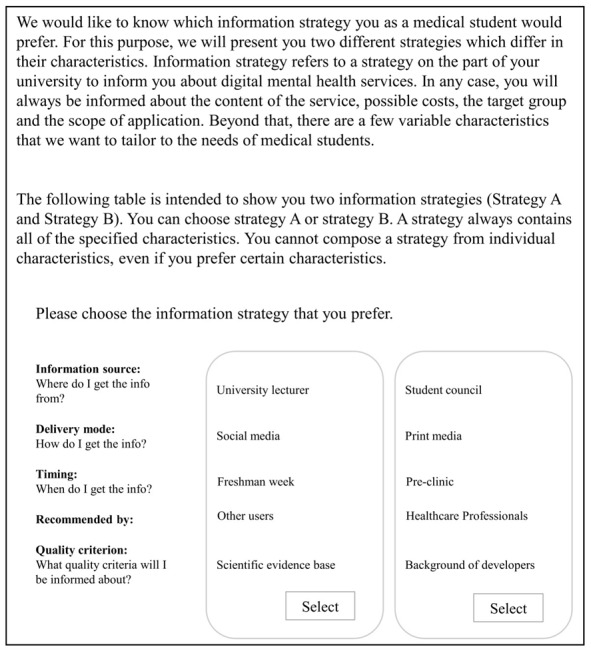
Instruction and example choice task of the discrete choice experiment to investigate preferences regarding information strategies for digital mental health interventions among medical students in Germany.

Participants were randomly assigned to 1 of the 3 blocks. In each block, we repeated 1 of the choice tasks to test the internal validity of the experiment as a reliability test. To familiarize respondents with the DCE format, we provided a detailed explanation for each attribute and attribute level (Table 1), followed by a written description of an example information strategy and an example choice task (Figure 1). Respondents were asked to choose the preferred information strategy to receive information about DMHIs. DMHIs were introduced as digital mental health services that can be used anonymously, from any location and at any time, for example, to reduce stress or combat examination anxiety. It was explained that DMHIs refer to smartphone apps (DiGA), browser-based applications, and psychological counseling via videoconferencing. We did not use an attribute for contents in this DCE due to anticipated heterogeneity in needs and knowledge [35].

**Table 1:** Final set of attributes and levels of the discrete choice experiment to investigate preferences regarding information strategies for DMHIs^a^ among medical students in Germany.

Attribute	Level 1	Level 2	Level 3	Level 4	Description
Information source	Student council^b^	Student services center	University lecturers	—^c^	Medical students could be informed on DMHIs by several organizational structures. Instances could be the student council, the student services center, and university lecturers.
Delivery mode	Email	Social media	Seminar	Print media	Medical students could receive information on DMHIs via different delivery modes. These include email, the social media accounts of the university, such as Instagram (Meta Platforms, Inc), a seminar that is included in the curriculum, and print media.
Timing	Freshman week^d^	Preclinic	Clinic	Practical year	Medical students could be informed about DMHIs by their university at different times. They could be informed right at the beginning of their studies during the freshman week, during the first stage of their studies (preclinic), during the clinical stage of their studies, or at the end of their studies during their practical year.
Recommendation	HCP^e^	Users	Students	No recommendation	Information strategies on DMHIs may contain reviews or recommendations. These could come from HCPs, such as physicians, other users, or students. They also may not contain statements about whether a service is recommended or advertised by other groups of people.
Quality criterion	Data security	Scientific evidence base	Background of developers	Quality seal	Medical students could receive information about quality criteria to help them decide whether DMHIs meet a certain standard. These include information on specific measures to safeguard data security, the scientific evidence base, or the professional background of the developers of a specific service. A quality seal informs if, for example, the university or a federal institute has received a quality certificate for its service.

^a^DMHI: digital mental health intervention.

^b^Student council: members of the student council are students who were elected to represent their perspective and interests in medical school.

^c^Not applicable.

^d^Freshman week: in Germany, student councils introduce freshmen to a variety of aspects of campus life over the course of the first week and provide orientation for medical studies. In this welcome week, many workshops and social activities are offered for new students.

^e^HCP: health care professional.

#### Technical Pretest

Finally, technical pretests of the first version of the programmed DCE with 3 feedback rounds (for iterative improvement) were conducted with 12 respondents from the institute of the principal investigator (eg, master students, doctoral candidates, research associates) and with 2 external medical students (personal contacts) in November and early December 2022. Technical pretesting resulted in several minor formal refinements of the survey interface. On the basis of pretest, the time required to complete the complete questionnaire (including the DCE and the questionnaire) was expected to be 15 to 20 minutes as planned.

Besides the DCE (including instructions), study information, and informed consent, the final survey asked for the background information presented in Textbox 1.

**Background information for the final survey.**

**Background information**

- Demographic characteristics (gender, age, and German state of the university)
- Studies (number of semesters, passed examinations—up to 3 in Germany until approbation)
- Familiarity with digital mental health interventions (DMHIs; if they have heard of DMHIs before)
- Attitudes toward potential use (how often they would like to use DMHIs: minutes a day and days a week)
- Willingness to pay for DMHIs (preferred mode of payment and amount of money willing to pay)
- Self-assessed stress in the previous and current semester using a visual analog scale (VAS) [42] ranging from 0 (minimum) to 10 (maximum)

### Recruitment and Data Collection

Medical students who stated that they were aged ≥18 years and enrolled at a medical school in Germany were able to participate in the anonymous web study by accessing a link to the survey website (eg, via QR code). We followed a convenience sampling strategy, which means participants were recruited via social media and email (via student councils), personal contacts (eg, face-to-face, WhatsApp [Meta Platforms, Inc] personal chat, and chat groups), printed flyers, and posters at different universities across Germany. We also involved lecturers to promote the study (showing slides with a QR code and link to the DCE in their presentation slides). We were also supported by the Study Dean of our Faculty of Medicine, including survey invitations sent via email to different semesters in April 2023.

Data collection took place from December 10, 2022, to May 2, 2023, once the required number of participants was reached. A sample size of >125 medical students was targeted to provide sufficient statistical power for the main analysis and several subgroup analyses based on a rule-of-thumb formula proposed by Johnson and Orme [43].

### Statistical Analysis

On the basis of the technical pretests of the web survey, it was assumed that a minimum processing time of 5 minutes was required to read the instructions and complete the DCE tasks properly; 5 minutes was a rather conservative estimation of the minimum processing time, as no participant in the pretest was able to complete the DCE in <5 minutes. For participants with lower processing time, we expected an insufficient engagement with the DCE (eg, choosing randomly and thereby increasing the error term of the analytical model) and excluded them from the analysis (ie, hard cutoff criterion). Moreover, participants who answered less than half of the choice sets (<4) were also excluded. Furthermore, the main analysis was based on respondents who passed the reliability test exclusively. The reliability test was passed if a repeated ninth control choice set was answered identically (ie, 1 choice set with strategy A and B swapped was repeated). Descriptive analysis for individual characteristics of the complete sample and participants that passed and did not pass the reliability test, respectively, were performed.

DCE data were analyzed using logistic regression models. In particular, we ran a conditional logit model clustered at the individual level to receive preference weights of levels as well as the relative importance of attributes. As the scale of coefficients is arbitrary, attributes are only comparable in relation to each other [44] using the relative importance of attributes.

In the second step, we considered possible preference heterogeneity by using a latent class analysis (LCA) model. The optimal class size was determined by the Bayesian information criterion and consistent Akaike information criterion. Classes were described by their preference weights and the relative importance of attributes. We also calculated posterior probabilities for being a member of a class for each participant. Respondents were assigned to the class with the higher posterior probability, and we compared individual characteristics of the assigned participants across classes descriptively. The analysis was performed with Stata 15 (StataCorp LLC). The significance level for statistical tests was an α error probability of <.05.

## Results

### Descriptive and Preliminary Analysis

The web survey was accessed 749 times. Of the 428 participants who gave informed consent, 97 (22.7%) needed a processing time of <5 minutes to complete the survey and were thus excluded from the analysis. Of those 97 participants who showed a processing time below 5 minutes, 66 (68%) participants did not complete at least 1 choice set, and another 5 (5%) respondents did complete <4 choice sets. Of the remaining 331 individuals, 309 (93%) respondents answered ≥4 choice sets of the DCE. In detail, those respondents who answered more than half of the choice sets completed all choice sets. Of those 22 individuals who answered <4 choice sets, 2 (9%) participants answered 2 choice sets, 1 (5%) participant answered one choice set, and 19 (86%) participants answered no choice set at all. The reliability test was passed by 231 (74.8%) of 309 participants (ie, complete samples). To describe choice-making behavior in the DCE in greater detail, we provide a lexicographic score analysis similar to Phillips et al [28], as shown in Multimedia Appendix 1 [28].

Characteristics of the sample are provided in Table 2 for the complete sample (N=309) as well as for participants who passed and did not pass the reliability test, respectively (n=231, 74.8 and n=78, 25.2%).

**Table 2:** Individual characteristics of the full study sample, a sample with participants who passed, and a sample with participants who did not pass the reliability test (passed if a repeated ninth control choice set was answered identically in the discrete choice experiment; N=309)^a^.

Variables		Full sample (N=309)	Passed reliability test (n=231)	Did not pass the reliability test (n=78)	Test^b^ (*P* value)	
**Gender, n (%)**						>.99^c^
	Women	217 (70.2)	162 (70.1)	55 (70.5)		
	Men	91 (29.4)	68 (29.4)	23 (29.5)		
	Nonbinary	1 (0.3)	1 (0.4)	0 (0)		
Age (y), mean (SD)		24.1 (4.0)	24.1 (3.9)	24.0 (4.1)	.88^d^	
Semesters, mean (SD)		7.1 (3.4)	7.2 (3.4)	7.0 (3.5)	.75^d^	
**Passed examinations, n (%)^e^**						.95^c^
	No passed examination	113 (36.6)	85 (36.8)	28 (35.9)		
	M1 examination	151 (48.9)	113 (48.9)	38 (48.7)		
	M2 examination	43 (13.9)	31 (13.4)	12 (15.4)		
	M3 examination	2 (0.6)	2 (0.9)	0 (0)		
**Self-assessed stress (previous semester), n (%)**						.32^c^
	0=“no stress at all”	0 (0)	0 (0)	0 (0)		
	1	11 (3.6)	10 (4.3)	1 (1.3)		
	2	8 (2.6)	7 (3)	1 (1.3)		
	3	33 (10.7)	24 (10.4)	9 (11.5)		
	4	25 (8.1)	15 (6.5)	10 (12.8)		
	5	21 (6.8)	18 (7.8)	3 (3.8)		
	6	31 (10)	19 (8.2)	12 (15.4)		
	7	61 (19.7)	46 (19.9)	15 (19.2)		
	8	62 (20.1)	48 (20.8)	14 (17.9)		
	9	34 (11)	28 (12.1)	6 (7.7)		
	10=“very stressed”	23 (7.4)	16 (6.9)	7 (9)		
**Self-assessed stress (currently), n (%)**						.33^c^
	0=“no stress at all”	23 (7.4)	17 (7.4)	6 (7.7)		
	1	24 (7.8)	20 (8.7)	4 (5.1)		
	2	47 (15.2)	36 (15.6)	11 (14.1)		
	3	37 (12)	23 (10)	14 (17.9)		
	4	25 (8.1)	16 (6.9)	9 (11.5)		
	5	26 (8.4)	19 (8.2)	7 (9)		
	6	27 (8.7)	19 (8.2)	8 (10.3)		
	7	39 (12.6)	32 (13.9)	7 (9)		
	8	31 (10)	22 (9.5)	9 (11.5)		
	9	13 (4.2)	11 (4.8)	2 (2.6)		
	10=“very stressed”	17 (5.5)	16 (6.9)	1 (1.3)		
Being aware of DMHIs^f^, n (%)		98 (31.7)	75 (32.5)	23 (29.5)	.68^d^	
Expected frequency of use (days per week), mean (SD)		2.2 (1.6)	2.2 (1.6)	2.3 (1.5)	.61^d^	
Expected frequency of use (minutes per day), mean (SD)		21.4 (16.5)	21.2 (15.5)	22.1 (19.2)	.60^d^	
Price participants were willing to pay (€^g^), mean (SD)		25.7 (60.4)	22.8 (22.0)	34.5 (114.2)	0.5^d^	

^a^No missing data in individual characteristics.

^b^Test for differences in individual characteristics for participants who passed the reliability test and those who did not pass the reliability test.

^c^Fisher exact test.

^d^Mann-Whitney U test.

^e^M1-M3: first to third state examination in medical schools in Germany.

^f^DMHI: digital mental health intervention.

^g^Exchange rate: 1€=US $1.11.

Most participants (217/309, 70.2%) in the full sample were women aged, on average, approximately 24 years (SD 4.0) and studying in their seventh semester (mean 7.1, SD 3.4). The mean age of men (mean 25.7, SD 4.4 years) was significantly higher than that of women (mean 23.3, SD 3.5 years) in the full sample (Mann-Whitney U test: *P*<.001), which also corresponds to a significantly higher mean semester for men than for women (mean 7.9, SD 3.1 vs mean 6.8, SD 3.5 semesters, Mann-Whitney U test: *P*=.006). Approximately one-third (113/309, 36.6%) of the university students in the complete sample passed no major examination to date, while nearly one-half (151/309, 48.9%) passed the M1 examination, which is the first state examination in medical studies and takes place after completion of the preclinical phase. The distribution of self-assessed stress during the previous semester was left-tailed, with a majority (211/309, 68.3%) of students indicating 6 to 10 on the 10-point rating scale, which might be interpreted as feeling moderately to severely stressed. In comparison to stress in the previous semester, the current self-assessed stress shifted to a lower level (only 127/309, 41.1% indicated a 6-10).

Of the 309 participants, 98 (31.7%) indicated being aware of DMHIs. On average, participants were willing to use DMHIs 2 days per week (mean 2.2, SD 1.6 days) and 21 minutes per day (mean 21.4, SD 16.5 min). As the most preferred payment mode, 93.2% (288/309) of students agreed on free offers. Nonetheless, the mean willingness to make a one-time payment for DMHI service was mean €25.7 (SD €60.4; 1€=US $1.11). A detailed analysis of the willingness to pay is provided in Multimedia Appendix 2.

Comparing individuals with a reliable versus a nonreliable response pattern according to their characteristics, no significant differences were detected (all with *P*>.05). In the following analysis, only participants who passed the reliability test were included. Yet, the results of the complete sample analysis are similar to the results based on the sample of respondents who passed the reliability test (Multimedia Appendix 3). Also, a Swait-Louviere test [45] indicated that while preferences were not significantly different for people who passed and did not pass the reliability test at the 5% significance level (H0a: *P*=.18), it also showed that the scale parameter differed significantly (H0b: *P*=.003).

### Main Findings

Table 3 presents the conditional logit regression results for respondents who passed the reliability test (n=231).

**Table 3:** Conditional logit regression results of the discrete choice experiment, showing significant attributes and levels as well as the relative importance of attributes in comparison.

Attributes and levels			Coefficient (SE; 95% CI^a^)		Relative importance (%)
**Information source**					9.22
	Student service center	–0.04 (0.05; –0.13 to 0.06)			
	Student council	0.17 (0.05; 0.07 to 0.28)			
	University lecturers	–0.14 (0.04; –0.22 to –0.05)			
**Delivery mode**					24.44
	Social media	0.27 (0.06; 0.15 to 0.39)			
	Email	0.16 (0.06; 0.05 to 0.27)			
	Seminar	0.12 (0.07; –0.02 to 0.25)			
	Print media	–0.55 (0.07; –0.68 to –0.42)			
**Timing**					32.59
	Freshman week	0.31 (0.06; 0.19 to 0.42)			
	Preclinic	0.50 (0.06; 0.38 to 0.62)			
	Clinic	–0.20 (0.05; –0.31 to –0.10)			
	Practical year	–0.60 (0.07; –0.73 to –0.47)			
**Recommendation**					19.09
	HCP^b^	0.14 (0.05; 0.04 to 0.24)			
	Students	0.29 (0.05; 0.18 to 0.39)			
	Users	–0.07 (0.05; –0.16 to 0.02)			
	No review	–0.35 (0.06; –0.47 to –0.24)			
**Quality criterion**					14.66
	Data security	–0.12 (0.06; –0.24 to –0.01)			
	Evidence based	0.30 (0.06; 0.18 to 0.41)			
	Quality seal	0.02 (0.05; –0.08 to 0.12)			
	Background of developers	–0.20 (0.06; –0.31 to –0.08)			

^a^CI significance is assumed if the CI does not include the number 0 (no different signs).

^b^HCP: health care professional.

Medical students significantly (*P*=.001) preferred to receive information about DMHIs from the student council and significantly refused to receive information from university lecturers (*P*=.002). As delivery mode, participants wanted to get the information delivered via social media (*P*<.001) and email (*P*=.006) and did not want to receive the information via print-based media (*P*<.001). Students had significant positive preference weights for receiving the information about DMHIs during their preclinic phase or in the freshman week and significant negative preference weights for the practical year (all with *P*<.001).

The recommendation should be given by other students (*P*<.001) or HCPs (*P*=.007), while no recommendation was rated negatively (*P*<.001).

As a quality criterion, medical students significantly preferred information about the evidence-based background of DMHIs (*P*<.001). Data security (*P*=.04) and background of developers (*P*=.001) showed significant negative preference weights.

With a relative importance of 32.6%, the most relevant attribute for medical students was timing (ie, time point in their studies when the information is shared). In addition, the attributes delivery mode (relative importance 24.4%) and recommendation (relative importance 19.1%) showed a high relative importance as well. The attributes of quality criterion (relative importance 14.7%) and information source (relative importance 9.2%) were less important for medical students.

To account for preference heterogeneity, we used an LCA. The results for respondents who passed the reliability test (n=231) are presented in Table 4. The optimal number of classes was determined by the Bayesian information criterion and consistent Akaike information criterion, which were minimized at 2 classes (Multimedia Appendix 4).

**Table 4:** Latent class model regression results of the discrete choice experiment, showing significant attributes and levels as well as the relative importance of attributes in comparison for 2 identified groups varying in information preferences (N=231).

Attributes and levels		Class 1 (n=147)				Class 2 (n=84)		
		Coefficient (SE; 95% CI)	Relative importance (%)		Coefficient (SE; 95% CI)		Relative importance (%)	
**Information source**				1.49				24.21
	Student service center	–0.03 (0.07; –0.18 to 0.11)			–0.07 (0.13; –0.32 to 0.18)			
	Student council	0.03 (0.07; –0.12 to 0.18)			0.56 (0.25; 0.07 to 1.04)			
	University Lecturers	0.00 (0.07; –0.14 to 0.14)			–0.49 (0.26; –0.99 to 0.02)			
**Delivery mode**				35.49				42.86
	Social media	0.09 (0.10; –0.10 to 0.29)			0.67 (0.19; 0.29 to 1.05)			
	Email	–0.09 (0.09; –0.27 to 0.08)			0.74 (0.29; 0.17 to 1.31)			
	Seminar	0.76 (0.13; 0.51 to 1.01)			–1.11 (0.38; –1.85 to –0.36)			
	Print media	–0.77 (0.11; –0.98 to –0.55)			–0.31 (0.49; –1.27 to 0.65)			
**Timing**				21.27				46.11
	Freshman week	0.14 (0.08; –0.02 to 0.29)			0.86 (0.32; 0.24 to 1.48)			
	Preclinic	0.48 (0.11; 0.27 to 0.69)			0.71 (0.31; 0.09 to 1.32)			
	Clinic	–0.18 (0.09; –0.36 to –0.003)			–0.43 (0.18; –0.78 to –0.09)			
	Practical year	–0.44 (0.10; –0.63 to –0.24)			–1.13 (0.46; –2.02 to –0.24)			
**Recommendation**				19.73				4.02
	HCP^a^	0.22 (0.09; 0.04 to 0.40)			–0.03 (0.33; –0.68 to 0.62)			
	Students	0.35 (0.09; 0.17 to 0.54)			0.04 (0.18; –0.32 to 0.39)			
	Users	–0.07 (0.08; –0.23 to 0.09)			0.08 (0.32; –0.55 to 0.72)			
	No review	–0.50 (0.06; –0.61 to –0.38)			–0.09 (0.45; –0.97 to 0.79)			
**Quality criterion**				22.02				19.10
	Data security	–0.39 (0.09; –0.56 to –0.22)			0.37 (0.26; –0.15 to 0.88)			
	Evidence based	0.56 (0.10; 0.35 to 0.76)			0.00 (0.18; –0.37 to 0.36)			
	Quality seal	–0.04 (0.08; –0.20 to 0.13)			0.09 (0.14; –0.19 to 0.38)			
	Background of developers	–0.13 (0.05; –0.23 to –0.03)			–0.46 (0.28; –1.00 to 0.08)			

^a^HCP: health care professional.

When assigning participants based on their posterior probabilities, we found that nearly two-thirds (147/231, 63.6%) could be allocated to class 1 (labeled “seminar-based information strategy”) and one-third (84/231, 36.4%) to class 2 (referred to as “early digital information strategy”). The 2 classes differed considerably in their preference patterns as well as in their relative importance of attributes. For class 1, the most important attribute was the delivery mode (approximately 36%) with seminar as the most preferred level. The attributes quality criterion, timing, and recommendation had similar shares of relative importance, approximately 20% each. In particular, preferences of class 1 showed that information about the evidence base of DMHIs should be used as a quality criterion, information should be provided during the preclinic phase, and recommendations for DMHIs should be given by students or HCPs. Information source was the least important attribute of class 1 (approximately 1%). According to the relative importance of attributes for class 2, timing (approximately 46%) and, with comparable relative importance, delivery mode (approximately 43%) were the most important attributes. Class 2 showed a strong preference for receiving the information about DMHIs early during their studies (preclinic phase or freshman week) and preferred receiving information via email or social media. The attribute information source showed a relative importance of approximately 24%, with student council as the preferred level. Regarding the attribute quality criterion (approximately 19%), data security was preferred by class 2 (not significant). The attribute recommendation was the least relevant attribute for class 2 (approximately 4%).

We compared the individual characteristics of members of class 1 and class 2, respectively, and found no significant differences (all with *P*<.05), except for being familiar with DMHIs (Multimedia Appendix 4). In detail, members of class 1 were significantly more familiar with DMHIs than members of class 2 (37.4% vs 23.8%).

## Discussion

### Principal Findings

In this study, we conducted a DCE to investigate information preferences regarding DMHIs for personal use among medical students in Germany. We aimed to derive a preferred information strategy and aimed to identify its most important features. In addition, we investigated whether there are latent classes among medical students that differ in information strategy preferences.

### Preferred Information Strategy and Relative Importance of Features

#### Overview

In the subsequent sections, the results regarding the various features of the preferred information strategy are discussed one by one. It is important to consider that participants thought about self-use, as indicated in the Introduction section. When addressing them as future physicians, results may be different.

#### Medical Students Preferred to Be Informed Early in the Freshman Week or the Preclinical Phase of Their Studies

Potentially, medical students (mean semester was 7, SD 3.4) had already experienced study-related mental health issues themselves or had seen such issues in their peers, which might lead to a preference for early information. Indeed, a meta-analysis indicated increased prevalence rates of mental health problems, especially in younger and preclinical students [16]. At best, the timing for information should be chosen when students feel mentally healthy or when academic distress has not already contributed to manifested health issues requiring interventions.

#### Information via Social Media and Email Was Favored Compared With Print-Based Media

Potentially, this reflects the habits and preferences of younger adults and digital natives included in our study. However, the research literature on the digital resource preferences of health care students belonging to the so-called Generation Z is interestingly limited and inconsistent [46]. In addition, in our study, participants even clearly refused to receive information via print media (eg, flyers). A possible explanation for this is the immense amount of printed information in medical schools. Students have to read and learn a great deal of complex content, mainly from textbooks, in a very short time for examination preparation. Possibly, they do not want the same modality of delivery that is regularly associated with study, work, or even stress for obtaining information concerning the promotion of their mental health. Another disadvantage of print media could be that it is visible to others when someone receives information via, for example, flyer or brochure. In contrast, brief information delivered digitally could be perceived as more pleasant (eg, a short video) and is easy to access whenever needed in daily life. Furthermore, the DCE was conducted about 3 years after the beginning of the COVID-19 pandemic, which led to the extension of e-learning in medical education. Elevated familiarity with digitally provided, approved health information in recent years may have increased the acceptance of digital information channels for other contexts [47]. Of course, students generally vary in learning preferences and learning styles [48] while research on learning styles in particular has been subject to controversy in terms of its evidence base and practical usefulness in medical education [49]. Therefore, we focused on stated preferences as well-researched constructs in the investigation of user-centered information strategies. However, we could only consider previously identified attributes and levels that may alter over time regarding their relevance for the target group with changing consumer habits and experiences. Future DCEs should explore which channel of social media (eg, Instagram [Meta Platforms, Inc] or TikTok [ByteDance]) and which presentation format (eg, video or participatory) should be used to disseminate information on DMHIs.

#### Participants Liked to Receive Recommendations From Other Students and HCPs

Both students and HCPs appeared suitable as role models (refer to social cognitive theory [50]) and trusted sources. Possibly, students preferred these sources as they are similar to themselves now (ie, as students) and hypothetically in the future (ie, as physicians), as different empirical investigations on the impact of narratives from individuals with a similar background on decision-making in health care contexts indicated [51,52]. Nonetheless, influences of recommendations are highly context sensitive.

For instance, in a DCE with young adults conducted by Cunningham et al [27], those who wanted to be informed digitally preferred recommendations by other young adults with previous experiences of depression and anxiety, while those who wanted to be informed conventionally via traditional media channels liked to receive recommendations from physicians. Hence, information features should be tailored to the preferences of the target group, including subgroups differing in information preferences (refer to LCA results mentioned subsequently). Although testimonials are commonly used in DMHI advertisements, it remains unclear how useful recipients view such recommendations as features of information or if the advertisements have an influence on the uptake of DMHIs [22]. Therefore, the integration of recommendations should be carefully chosen in dialog with the key target group for the information distribution. In future studies, it would be interesting to investigate the characteristics of recommendations that students perceive as trustworthy (eg, those with own experience with mental health issues).

#### Regarding Quality Criteria, Medical Students Significantly Preferred Information About the Evidence-Based Background of DMHIs

Data security and background of developers showed significant negative preference weights. Students expected the information to be provided by the university (as stated in the DCE’s instructions), and possibly assumed that the offerings were already quality tested and therefore focused on other quality criteria than they would be interested in another setting. Addressed in their role as future professionals, preferences might differ, and participants may place more emphasis on information on the evidence base or data security for professional use (eg, refer to studies by Braun et al [35] and Dederichs et al [36]).

#### Medical Students Preferred to Receive Information About DMHIs From Their Student Council

Medical students even refused to receive information from university lecturers. With respect to mental health topics, students may have a higher trust in students’ representatives as peers and may worry about stigmatization by lecturers if they disclose their need for support.

### Subgroup-Specific Information Strategies

LCA identified 2 distinct groups of students who differed considerably in their information preferences. Class 1, which we classified as “seminar-based information strategy,” preferred to receive information about DMHIs in a face-to-face seminar in the preclinic phase. Moreover, they preferred to obtain recommendations from other students, while information about the evidence base of DMHIs should serve as a key quality criterion. Except for the delivery mode (seminar), preferences of class 1 were similar to the overall preferences of participants regarding DMHI information strategies, which can be well explained by the fact that the majority (ie, 147/231, 63.6%) could be classified as members of class 1.

In contrast, the ideal information strategy of class 2 (“early digital information strategy”) would be to provide information by the student council via social media or by email. Regarding the timing of information, class 2 preferred to receive them as early as possible, that is, during the freshman week (before the actual start of studies). The attributes quality criteria and recommendation were less important for class 2 than for class 1, and the findings were insignificant. Members of class 2 were significantly less familiar with DMHIs than members of class 1. Hence, they may want to be initially informed at a low threshold. In prior qualitative studies with medical students, they expressed the wish to get quick support by interventions that are easy to access in times of study-related distress, for example, services targeting stress and time management [36,53]. Furthermore, we did not find any significant differences (eg, in stress levels) in the individual characteristics of members of class 1 and class 2.

However, preferences of classes 1 and 2 should be not viewed as divergent strategies but may help to develop complementary stepped or matched approaches. For instance, digitally provided information could be the first step that might lead to further information-seeking behavior and the attendance of seminars on DMHIs. A combination of both strategies should be used to reach a higher number of medical students throughout Germany. Specifically, the digital strategy, which is in line with the overall preference, represents a way to reach a broad range of medical students, especially those with little knowledge or no currently perceived need for DHMIs.

Besides the DCE, our study provided new insights into the willingness to pay for DMHIs, which should also be considered by universities when informing about interventions. In particular, medical students stated being willing to pay €22.7 (US $ 25.25) as a unique fee on average to use DMHIs.

### Limitations

A key limitation was that we recruited medical students mainly via one channel, namely, personal invitations (eg, chat groups and emails from student councils), as postings in social media groups (eg, on Facebook [Meta Platforms, Inc]), as well as flyers on campus across different universities, were much less effective in terms of recruitment success. Nonetheless, the sample seems to be similar to the overall population of medical students in Germany regarding their individual characteristics, such as age and gender (eg, study by Groene et al [54]).

Another limitation was that students could participate during the semester and during the semester break. Information preferences regarding mental health services might differ depending on the time of participation and related needs and stress levels (eg, in times of examinations and semester appointments). Specifically, increased perceived stress may indicate personal need or relevance of mental health information and thereby foster the motivation to seek information on coping strategies, which may be related to more interest in easily accessible support services, such as DMHIs.

However, our study can only provide very limited information on whether or how emotional distress may have influenced DMHI information preferences. Due to ethical aspects and to keep the (emotional) response burden as low as possible, we refrained from using clinical screening measures on CMDs in this study. A future DCE could put a greater emphasis on information preference on DMHIs for self-help and adjunctive treatment, which could include the assessment of different mental health conditions.

A further limitation was that most participants had not heard of DMHIs before, which is similar to prior studies with students from our work group [25,36,55,56] but may have resulted in difficulties in decision-making and thus contributed to heuristic decisions (eg, finding student councils more appealing than lectures and thus wanting them as information source).

It should be noted that there was no opt-out option given to respondents, which corresponds to the real-world setting of students receiving information by a university information campaign. However, our results may be biased to some extent as students not wanting to receive information may differ in their preferences from those wanting to receive information.

Finally, we will discuss a few thoughts on the selected attributes and levels. Concerning the level “seminar,” belonging to the attribute “delivery mode,” further information on the organization of a seminar (eg, block seminar, face-to-face seminar, and web seminar) would be desirable. Further research should clarify the preferences of medical students regarding the design of seminars about DMHIs (eg, mode, duration, and topic).

Finally, there might be a lack of selectivity in the attribute levels “students” and “users” regarding the attribute “recommendation” because students can also be users at the same time. However, due to the different assessments (with other students being a significant recommendation source), it seems that the participants viewed those attributes differently, and therefore, this limitation may not be severe. Future DCEs could use additional qualitative methods to prevent such potential sources of bias (eg, by more detailed descriptions of prototypical users or personas to be developed with the target group).

### Conclusions

Overall, the study findings suggest distinct general and subgroup-specific information strategies on DMHIs that are potentially suitable for medical students in Germany. Designing information strategies according to the stated preferences could help to increase DMHI use. This DCE offers a variety of possible features to be tested in practice. As the first step, AFI strategies could start to promote the awareness of or basic knowledge of DMHIs for health promotion purposes with a social media campaign related to the university setting aimed at students early in their studies. The campaign could be provided by the student council, combining recommendations and information about scientific evidence as complementary features. This low-threshold offer could lead to students attending seminars on DMHIs involving more detailed knowledge in a second step. Seminars should be firmly anchored in the curricula of the medical faculties in accordance with the mandatory German National Competency-Based Learning Objective Catalogue of Medicine; digital competencies have not been listed for very long in this catalogue [57], but are now, albeit to a limited extent.

## Appendix

Multimedia Appendix 1 Choice-making behavior–lexicographic scores.

Multimedia Appendix 2 Willingness to pay for digital mental health interventions.

Multimedia Appendix 3 Results of the complete sample analysis.

Multimedia Appendix 4 Further information on latent class analysis.

## Abbreviations

AFI: acceptance-facilitating intervention
BfARM: Bundesinstitut für Arzneimittel und Medizinprodukte
CMD: common mental disorder
DCE: discrete choice experiment
DiGA: Digitale Gesundheitsanwendungen
DMHI: digital mental health intervention
HCP: health care professional
LCA: latent class analysis

## Data Availability

The data sets generated during and analyzed during this study are available from the corresponding author on reasonable request.
