# Supplementary material for: Preferences Regarding Information Strategies for Digital Mental Health Interventions Among Medical Students: Discrete Choice Experiment
Source: JMIR Form Res. 2024 Oct 4;8:e55921. doi: 10.2196/55921 (PMC11489795; doi:10.2196/55921)
Supplement: Multimedia Appendix 1 [file formative_v8i1e55921_app1.pdf]

Multimedia Appendix

This is a Multimedia Appendix to a full manuscript published in JMIR Mental Health. For full copyright and citation information see http://dx.doi.org/10.2196/jmir.55921.

Multimedia Appendix 1: Choice making behavior – lexicographic scores.

To study choice making behavior in greater detail, we calculate lexicographic scores similar to Phillips et al. (2021). In particular, we counted for each level within an attribute the number of actual choices that include this level. This number was divided by the number of choice-sets including the level under consideration to calculate lexicographic scores. According to Phillips et al. (2021) a respondent was categorized as having lexicographic behavior if the lexicographic score was at least 90%.

Table S1 shows how often a choice-set includes the respective level. Choice-sets in the discrete choice experiment (DCE) never include the same level in both alternatives. All nine choice-sets were considered since the two choice-sets constituting the reliability test are informative to detect lexicographic behavior. The table also states the number of respondents who shows lexicographic scores of at least 90%. For all levels we found respondents with a lexicographic score of at least 90%. A bigger proportion of individuals across all blocks showed lexicographic scores of at least 90% for social media regarding delivery mode, freshmen week and pre-clinic regarding timing, students regarding recommendation, and scientific evidence regarding quality criterion.

Only 53 (17.15%) out of all 309 respondents showed lexicographic scores lower than 90% for all levels (Table S2). The majority of individuals had lexicographic scores equal or above 90% in one or two levels. Several respondents were detected to have lexicographic scores equal or above 90% in up to four levels.

Our experimental design constitutes limitation to the analysis of lexicographic behavior. The number of choice-sets including a certain level varied between two and seven choice-sets. Especially for levels available in only few choice-sets, respondents may choose the level by accident rather than by lexicographic behavior. Furthermore, the number of total choice-sets is too small to detect lexicographic behavior reliably in our DCE. Note that the criteria to detect lexicographic behavior may be too strong as a lexicographic score of 90% is equivalent to choosing the level in all possible cases in our experimental design. While results are inconclusive to detect lexicographic behavior reliably, it provides deeper insights in decision making in our DCE. We can infer that levels like social media for delivery mode, freshmen week and pre-clinic for timing, students for recommendation, and scientific evidence for quality criterion are relevant for the decision making process for many respondents.

Table S1: Overview of individuals identified as having lexicographic behavior, showing the number of choice-sets with level available in at least one alternative and the number of participants with lexicographic score of at least 90%, within the framework of a discrete choice experiment to investigate preferences regarding information strategies for digital mental health interventions among medical students in Germany.

**Attribute / Level**	**Number of choice-sets with level available in at least one alternative**			**Number of participants with lexicographic score of at least 90%**			
	**Block 1**	**Block 2**	**Block 2**	**Block 1 (N=104)**	**Block 2 (N = 88)**	**Block 2 (N = 117)**	**Total (N = 309)**
Information source							
Student services centre	7	6	6	3	1	1	5 (1.62%)
Student council	6	5	7	1	9	13	23 (7.44%)
University lecturers	5	7	5	1	1	3	5 (1.62%)
Delivery mode							
Social media	5	4	5	5	8	22	35 (11.33%)
E-mail	4	4	4	9	11	5	25 (8.09%)
Seminar	5	5	5	5	10	10	25 (8.09%)
Print media	4	5	4	3	0	2	5 (1.62%)
Timing							
Freshman week	5	5	4	5	12	22	39 (12.62%)
Pre-clinic	4	4	5	22	8	20	50 (16.18%)
Clinic	4	5	5	2	4	0	6 (1.94%)
Practical year	5	4	4	8	0	2	10 (3.24%)
Recommendation							
HCP[a]	5	4	5	1	5	14	20 (6.47%)
Students	7	3	3	0	33	5	38 (12.30%)
Users	4	5	4	3	0	8	11 (3.56%)
No recommendation	2	6	6	21	0	0	21 (6.80%)
Quality criterion							
Data security	6	4	5	3	0	2	5 (1.62%)
Scientific evidence base	3	4	6	11	24	4	39 (12.62%)
Quality seal	4	4	4	7	3	17	27 (8.74%)

Background of developers	5	6	3	1	4	0	5 (1.62%)

[a]HCP = healthcare professionals.

Table S2: Distribution of individuals according to the number of levels with lexicographic score ≥ 90% within the framework of a discrete choice experiment to investigate preferences regarding information strategies for digital mental health interventions among medical students in Germany.

**Number of levels with lexicographic scores ≥ 90%**	**Respondents (N = 309)**
0	53 (17.15%)
1	140 (45.31%)
2	97 (31.39%)
3	16 (5.18%)
4	3 (0.97%)

References

Elena A. Phillips et al. 2021 Preferences for e-Mental Health Interventions in Germany: A Discrete Choice Experiment, Value in Health, Volume 24, Issue 3, 421 - 430
