# Supplementary material for: Preferences Regarding Information Strategies for Digital Mental Health Interventions Among Medical Students: Discrete Choice Experiment
Source: JMIR Form Res. 2024 Oct 4;8:e55921. doi: 10.2196/55921 (PMC11489795; doi:10.2196/55921)
Supplement: Multimedia Appendix 2 [file formative_v8i1e55921_app2.pdf]

## Multimedia Appendix

This is a Multimedia Appendix to a full manuscript published in JMIR Mental Health. For full copyright and citation information see http://dx.doi.org/10.2196/jmir.55921.

## Multimedia Appendix 2: Willingness to pay for digital mental health interventions (DMHIs).

The payment mode was assessed in the online survey questionnaire by asking participants to state which payment mode is applicable to them. Participants had to tick the categories which they evaluate applicable. Multiple answers were allowed for respondents. Table S3 shows the possible payment modes and the fraction of participants who stated that they evaluate the payment mode applicable to them.

Table S3: Payment mode for DMHIs ordered according to the applicability for medical students in Germany.[a]

| **Payment Mode** | **Fraction of participants who stated that the payment mode were applicable to them** |
|---|---|
| | |
| No payment | 93.20% |
| Payment within the semester fee | 72.49% |
| Unique payment | 47.25% |
| Unique payment per package of DMHI | 38.19% |
| Subscription with monthly payments | 21.40% |
| Subscription with yearly payments | 5.50% |

[a]n= 309 participants. Multiple selections of payment modes were possible.

No payment was acceptable for nearly all students. The second best option with a 72.49% agreement rate was payment within the semester fee. Roughly half of the students considered an unique payment as acceptable. Unique payment per package of the DMHI, and subscription with payments on monthly or yearly basis are less acceptable for students.

The willingness to pay (WTP) regarding electronic mental health interventions (DMHIs) ranged between 0 and 150 for participants who passed the reliability test. The mean WTP was € 22.76 (median WTP € 20.00) with a standard deviation of 21.99. Out of the 231 participants of the sample, 13 (5.63%) participants had a WTP of zero. The distribution of the WTP is right-tailed (Figure S1).

Figure S1: Distribution of the willingness to pay for DMHIs among medical students in Germany.

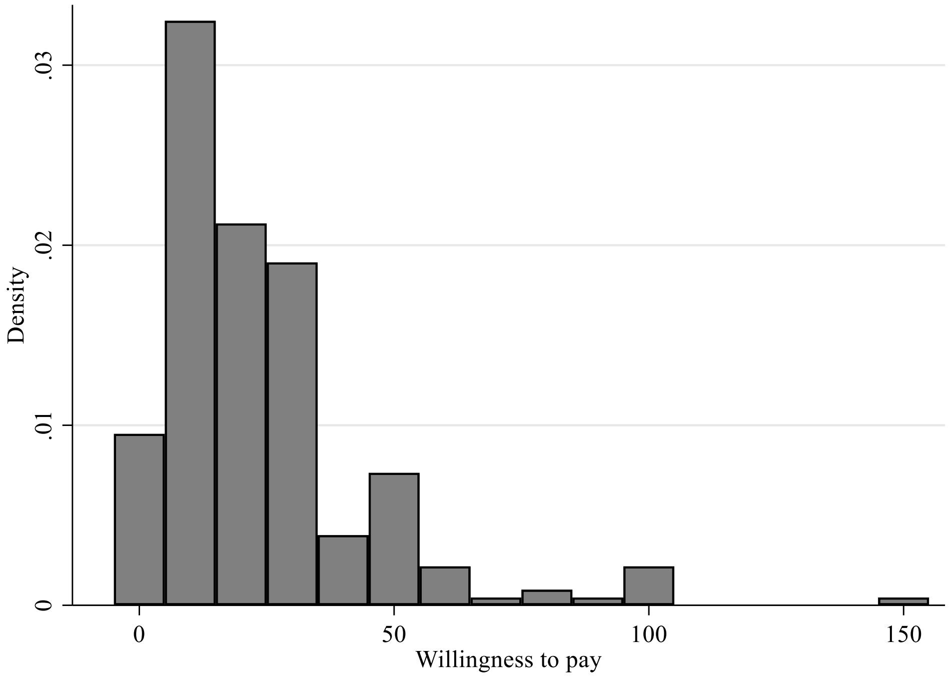


We provide information about the demand for DMHIs with respect to prices, we calculated the demand curve (Figure S2). The dotted line indicates the mean willingness to pay of € 22.76. For a unique payment of € 22.76, 36.80% of the participants would be willing to pay for the DMHIs. For a price of € 20.00 (the median WTP), 53.25% of the students would be willing to pay the DMHIs and roughly three-third of all participants (75.32%) would be willing to pay for the DMHIs for € 10.00.

Figure S2: Demand curve for DMHIs among medical students in Germany, indicating the mean willingness to pay (dotted line).

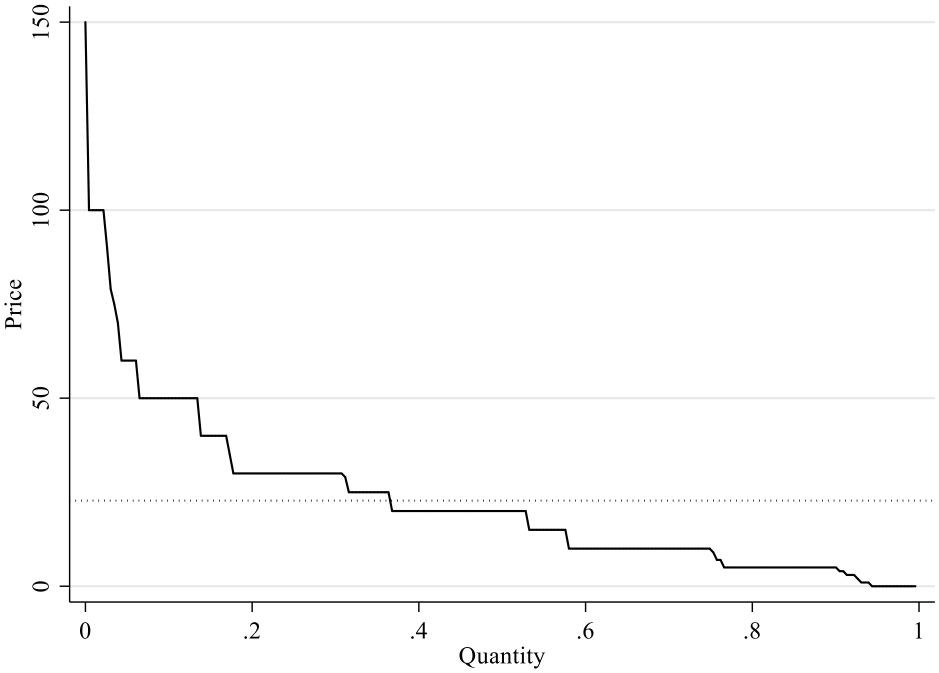


To analyze the influence of individual characteristics and attitudes towards DMHIs on the WTP, we estimated an OLS regression. The person with non-binary / third gender was excluded from the analysis. Results of the OLS regression (Table S4) showed that individual characteristics and attitudes towards DMHIs did not have a significant effect on WTP for DMHIs except for expected frequency of use in minutes per day. In particular, a higher expected use of DMHIs by one minute per day increases the willingness to pay by approximately € 0.60.

Table S4: OLS regression results on the willingness to pay for DMHIs among medical students in Germany, controlling for the influence of individual characteristics and attitudes towards DMHIs.

| | Specification (1) | Specification (2) | Specification (3) |
|---|---|---|---|
| Gender (1 = Men) | -1.838<br>(7.573) | -2.246<br>(7.776) | -1.882<br>(7.792) |
| Semesters | -1.029<br>(2.203) | | |
| Age | | 0.0406<br>(1.005) | -6.379<br>(7.678) |
| $Age^2$ | | | 0.118<br>(0.139) |

| | | | |
|---|---|---|---|
| *Passed exams* | | | |
| M1 | -2.416 | -7.193 | -4.966 |
| | (12.54) | (8.088) | (8.512) |
| M2 | 1.691 | -7.377 | -3.976 |
| | (21.88) | (11.64) | (12.32) |
| M3 | -3.741 | -12.51 | -8.363 |
| | (46.62) | (42.88) | (43.19) |
| Familiar with DMHIs (1 = being familiar) | -6.309 | -6.426 | -6.325 |
| | (7.420) | (7.440) | (7.444) |
| Expected frequency of use - days per week, mean | 3.094 | 3.166 | 3.147 |
| | (2.199) | (2.203) | (2.204) |
| Expected frequency of use - minutes per day | 0.603** | 0.615** | 0.627** |
| | (0.216) | (0.215) | (0.216) |
| Constant | 16.75 | 11.85 | 94.44 |
| | (12.38) | (23.19) | (100.6) |
| *N* | 308 | 308 | 308 |
| $R^2$ | 0.049 | 0.048 | 0.051 |

OLS Regression on willingness to pay for DMHIs. Standard errors in parentheses. * $p < 0.05$, ** $p < 0.01$, *** $p < 0.001$
