# Supplementary material for: Preferences Regarding Information Strategies for Digital Mental Health Interventions Among Medical Students: Discrete Choice Experiment
Source: JMIR Form Res. 2024 Oct 4;8:e55921. doi: 10.2196/55921 (PMC11489795; doi:10.2196/55921)
Supplement: Multimedia Appendix 3 [file formative_v8i1e55921_app3.pdf]

Multimedia Appendix

This is a Multimedia Appendix to a full manuscript published in JMIR Mental Health. For full copyright and citation information see http://dx.doi.org/10.2196/jmir.55921.

Multimedia Appendix 3: Results of the complete sample analysis.

Table S5: Conditional logit regression results for the complete sample of the discrete choice experiment, showing significant attributes and levels as well as the relative importance of attributes in comparison.[a]

Attributes and Levels	Coefficient (SE)[b]	95% CI[c]	Relative Importance
**Information Source**			8.29%
Student Service Centre	-0.02 (0.04)	[-0.10, 0.06]	
Student Council	0.14 (0.04)	[0.05, 0.22]	
University Lecturers	-0.12 (0.04)	[-0.20, -0.04]	
**Delivery Mode**			24.21%
Social Media	0.23 (0.05)	[0.13, 0.33]	
E-Mail	0.13 (0.05)	[0.04, 0.22]	
Seminar	0.15 (0.06)	[0.04, 0.26]	
Print Media	-0.52 (0.06)	[-0.62, -0.41]	
**Timing**			31.48%
Freshman Week	0.26 (0.05)	[0.16, 0.35]	
Pre-Clinic	0.43 (0.05)	[0.33, 0.53]	
Clinic	-0.14 (0.05)	[-0.23, -0.06]	
Practical Year	-0.54 (0.06)	[-0.65, -0.43]	
**Recommendation**			20.88%
HCP[d]	0.19 (0.04)	[0.11, 0.28]	
Students	0.26 (0.05)	[0.17, 0.35]	
Users	-0.07 (0.04)	[-0.15, 0.005]	
No Review	-0.38 (0.05)	[-0.48, -0.29]	
**Quality Criterion**			15.14%
Data Security	-0.09 (0.05)	[-0.19, 0.01]	
Evidence-Based	0.27 (0.05)	[0.17, 0.36]	

Quality Seal	0.03 (0.05)	[-0.06, 0.12]	
Background of Developers	-0.20 (0.05)	[-0.30, -0.10]	

[a]Regression is estimated without repeated choice-set 9. Results based on n=309.
[b]SE = standard error.
[c]CI = confidence interval.
[d]HCP = healthcare professionals.

Table S6: Latent class model regression results for the complete sample of the discrete choice experiment, showing significant attributes and levels as well as the relative importance of attributes in comparison for two identified groups varying in information preferences.[a]

**Attributes and Levels**	**Class 1**			**Class 2**		
	Coefficient (SE)[b]	95% CI[c]	Relative Importance	Coefficient (SE)	95% CI	Relative Importance
**Information Source**			11.17%			5.70%
Student Service Centre	-0.21 (0.10)	[-0.41, -0.004]		0.10 (0.07)	[-0.03, 0.23]	
Student Council	0.34 (0.10)	[0.15, 0.54]		0.08 (0.07)	[-0.04, 0.21]	
University Lecturers	-0.14 (0.09)	[-0.31, 0.04]		-0.18 (0.07)	[-0.33, -0.04]	
**Delivery Mode**			29.21%			28.24%
Social Media	0.68 (0.13)	[0.43, 0.93]		-0.01 (0.11)	[-0.23, 0.22]	
E-Mail	0.47 (0.14)	[0.20, 0.75]		-0.14 (0.09)	[-0.31, 0.03]	
Seminar	-0.77 (0.22)	[-1.19, -0.34]		0.77 (0.14)	[0.50, 1.04]	
Print Media	-0.39 (0.12)	[-0.63, -0.14]		-0.63 (0.09)	[-0.80, -0.45]	
**Timing**			35.66%			13.75%
Freshman Week	0.43 (0.11)	[0.22, 0.65]		0.17 (0.08)	[0.02, 0.32]	
Pre-Clinic	0.79 (0.15)	[0.50, 1.09]		0.35 (0.09)	[0.18, 0.53]	
Clinic	-0.26 (0.11)	[-0.47, -0.05]		-0.19 (0.09)	[-0.37, -0.02]	
Practical Year	-0.97 (0.15)	[-1.26, -0.69]		-0.33 (0.11)	[-0.55, -0.10]	
**Recommendation**			8.06%			23.67%
HCP[d]	0.25 (0.09)	[0.06, 0.43]		0.20 (0.07)	[0.06, 0.34]	
Students	-0.14 (0.13)	[-0.40, 0.12]		0.51 (0.10)	[0.31, 0.72]	
Users	-0.15 (0.11)	[-0.37, 0.06]		-0.05 (0.07)	[-0.19, 0.09]	

No Review	0.05 (0.18)	[-0.30, 0.40]		-0.66 (0.11)	[-0.88, -0.45]	
**Quality Criterion**			15.91%			18.01%
Data security	0.20 (0.15)	[-0.10, 0.51]		-0.34 (0.10)	[-0.53, -0.15]	
Evidence-Based	-0.11 (0.13)	[-0.36, 0.14]		0.55 (0.10)	[0.35, 0.75]	
Quality Seal	0.35 (0.13)	[0.09, 0.60]		-0.20 (0.11)	[-0.40, 0.01]	
Background of Developers	-0.44 (0.11)	[-0.66, -0.22]		-0.02 (0.08)	[-0.18, 0.15]	

[a]Bayesian Information Criterion and consistent Akaike Information Criterion were minimized at two classes. Regression is estimated without repeated choice-set 9. Results based on n=309.
[b]SE = standard error.
[c]CI = confidence interval.
[d]HCP = healthcare professionals.
