# Supplementary material for: Preferences Regarding Information Strategies for Digital Mental Health Interventions Among Medical Students: Discrete Choice Experiment
Source: JMIR Form Res. 2024 Oct 4;8:e55921. doi: 10.2196/55921 (PMC11489795; doi:10.2196/55921)
Supplement: Multimedia Appendix 4 [file formative_v8i1e55921_app4.pdf]

Multimedia Appendix

This is a Multimedia Appendix to a full manuscript published in JMIR Mental Health. For full copyright and citation information see http://dx.doi.org/10.2196/jmir.55921.

Multimedia Appendix 4: Further information on latent class analysis.

Table S7: Information criteria for different number of classes in a latent class analysis within the framework of a discrete choice experiment to investigate preferences regarding information strategies for digital mental health interventions among medical students in Germany.[a]

No. of classes	BIC[b]	cAIC[c]
2	2234.2606	2263.2606
3	2260.7319	2304.7319
4	2304.1565	2363.1565
5	2352.8563	2426.8563

[a]Results are based on participants who passed the reliability test.
[b]BIC = Bayesian information criterion.
[c]cAIC = consistent Akaike Information Criterion.

Table S8: Individual characteristics of participants who passed the reliability test (passed if a repeated ninth control choice set was answered identically in the discrete choice experiment) for class 1 and class 2, identified in a latent class analysis.[a]

Variable	Class 1 (N = 147)	Class 2 (N = 84)	Test[b] (p-value)
Gender			0.508[c]
Women, N (share)	104 (70.75%)	58 (69.05%)	
Men, N (share)	43 (29.25%)	25 (29.76%)	
Non-binary / third gender, N (share)	0 (0.00%)	1 (1.19%)	
Age, mean (SD)[e]	24.12 (3.70)	23.94 (4.28)	0.350[d]
Semesters, mean (SD)	7.20 (3.33)	7.11 (3.47)	0.966[d]
Passed exams			0.869[c]
No passed exam, N (share)	52 (35.37%)	33 (39.29%)	
M1, N (share)	73 (49.66%)	40 (47.62%)	
M2, N (share)	21 (14.29%)	10 (11.90%)	
M3, N (share)	1 (0.68%)	1 (1.19%)	
Self-assessed stress - previous semester			0.730[c]
0 = "no stress at all", N (share)	0 (0.00%)	0 (0.00%)	
1, N (share)	7 (4.76%)	3 (3.57%)	
2, N (share)	4 (2.72%)	3 (3.57%)	
3, N (share)	16 (10.88%)	8 (9.52%)	
4, N (share)	10 (6.80%)	5 (5.95%)	
5, N (share)	14 (9.52%)	4 (4.76%)	
6, N (share)	15 (10.20%)	4 (4.76%)	
7, N (share)	26 (17.69%)	20 (23.81%)	
8, N (share)	30 (20.41%)	18 (21.43%)	
9, N (share)	15 (10.20%)	13 (15.48%)	

10 = "very stressed", N (share)	10 (6.80%)	6 (7.14%)	
Self-assessed stress - currently			0.085[c]
0 = "no stress at all", N (share)	11 (7.48%)	6 (7.14%)	
1, N (share)	12 (8.16%)	8 (9.52%)	
2, N (share)	21 (14.29%)	15 (17.86%)	
3, N (share)	18 (12.24%)	5 (5.95%)	
4, N (share)	14 (9.52%)	2 (2.38%)	
5, N (share)	15 (10.20%)	4 (4.76%)	
6, N (share)	11 (7.48%)	8 (9.52%)	
7, N (share)	19 (12.93%)	13 (15.48%)	
8, N (share)	10 (6.80%)	12 (14.29%)	
9, N (share)	9 (6.12%)	2 (2.38%)	
10 = "very stressed", N (share)	7 (4.76%)	9 (10.71%)	
Familiar with DMHIs, N (share)	55 (37.41%)	20 (23.81%)	0.041[c]
Expected frequency of use - days per week, mean (SD)	2.31 (1.63)	2.01 (1.59)	0.110[d]
Expected frequency of use - minutes per day, mean (SD)	20.61 (15.54)	22.26 (15.55)	0.460[d]
Willingness to pay, mean (SD)	21.78 (21.13)	24.48 (23.43)	0.631[d]

[a]No missing data in individual characteristics.

[b]Test for differences in individual characteristics for participants of class 1 and class 2.

[c]Fisher's exact test.

[d]Mann-Whitney-U test.

[e]SD = standard deviation.
